# The Complex Relationship between Virulence and Antibiotic Resistance

**DOI:** 10.3390/genes8010039

**Published:** 2017-01-18

**Authors:** Meredith Schroeder, Benjamin D. Brooks, Amanda E. Brooks

**Affiliations:** 1 Department of Microbiological Sciences; North Dakota State University, Fargo, ND 58105, USA; meredith.irsfeld.1@ndsu.edu; 2 Department of Electrical and Computer Engineering; North Dakota State University, Fargo, ND 58105, USA; ben@brooks.nu; 3 Department of Pharmaceutical Sciences, North Dakota State University, Fargo, ND 58105, USA

**Keywords:** antibiotic resistance, virulence genes, biofilms, microbial communication

## Abstract

Antibiotic resistance, prompted by the overuse of antimicrobial agents, may arise from a variety of mechanisms, particularly horizontal gene transfer of virulence and antibiotic resistance genes, which is often facilitated by biofilm formation. The importance of phenotypic changes seen in a biofilm, which lead to genotypic alterations, cannot be overstated. Irrespective of if the biofilm is single microbe or polymicrobial, bacteria, protected within a biofilm from the external environment, communicate through signal transduction pathways (e.g., quorum sensing or two-component systems), leading to global changes in gene expression, enhancing virulence, and expediting the acquisition of antibiotic resistance. Thus, one must examine a genetic change in virulence and resistance not only in the context of the biofilm but also as inextricably linked pathologies. Observationally, it is clear that increased virulence and the advent of antibiotic resistance often arise almost simultaneously; however, their genetic connection has been relatively ignored. Although the complexities of genetic regulation in a multispecies community may obscure a causative relationship, uncovering key genetic interactions between virulence and resistance in biofilm bacteria is essential to identifying new druggable targets, ultimately providing a drug discovery and development pathway to improve treatment options for chronic and recurring infection.

## 1. Introduction

Until recently, conventional “antibiotic wisdom” suggesting the presence of a fitness cost associated with the development of antibiotic resistance that would eventually allow susceptible species to overtake resistant species was the predominating dogma in infectious diseases [1]. However, the ever-increasing threat of antibiotic resistant bacteria contradicts dogma and insinuates that the evolution of resistance may be associated with a fitness advantage, including enhanced virulence [2,3]. Although virulence has now been directly related to multidrug resistance in several animal infection models [2], the mechanism of virulence regulation in this climate of antibiotic resistance remains elusive. This review will explore the relationship between the mechanisms of acquired antibiotic resistance and enhanced virulence, a critical link in our war on the emergence of multidrug resistant bacteria.

## 2. Antibiotic Resistance

**Figure 1:**
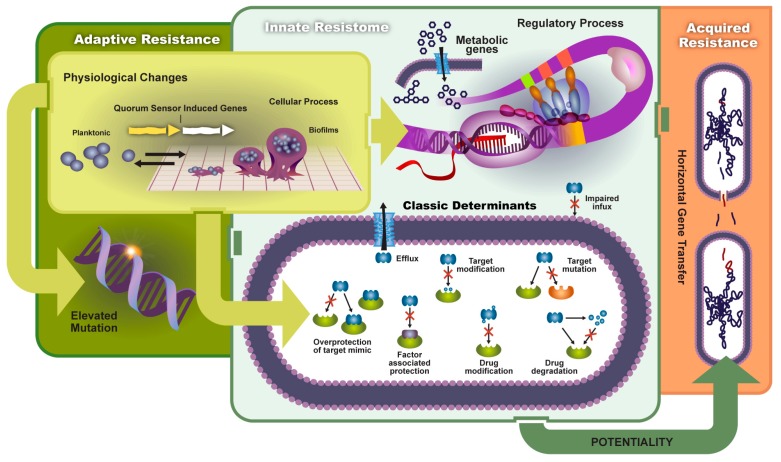
The transmission and mechanisms of antibiotic resistance and virulence can be divided into adaptive resistance, innate resistance, and acquired resistance. Environmental factors can prompt physiological changes and lead to: (1) elevated mutation rates; (2) changes in metabolic genes and regulatory processes; and (3) a host of classic antibiotic inactivation and resistance mechanisms (classic determinants). Such resistance and increased virulence can potentially be shared among bacteria leading to acquired resistance.

The introduction of antibiotics over half a century ago revolutionized medicine. Although antibiotics are fundamentally effective against bacteria [4,5,6], compound development is locked in a co-evolutionary battle with natural bacterial compounds, inadvertently fostering the development of resistance. Bacteria have found a host of mechanisms to circumvent antibiotic killing and transmit resistance [7,8,9,10], that until recently were thought to impose a significant burden on overall evolutionary fitness [1], allowing susceptible organisms to ultimately outcompete their resistant counterparts. Generally, these mechanisms can be grouped as either innate resistance or, of more concern to modern clinical and agriculture practice, acquired resistance [11,12,13,14] (Figure 1). Unfortunately, the once predominant dogma that described antibiotic resistance and fitness as an inverse relationship has not been borne out in the face of ever-increasing reports of antibiotic resistant bacteria, hinting at the possibility of compensating co-mutations.

### 2.1. Types of Resistance

#### 2.1.1. Innate Resistance

Genes encoding antimicrobial resistance inherently present in bacteria that produce antibiotics confer innate resistance. Davies et al. posit the hypothesis that the spread in antibiotic resistance may be a consequence of antibiotic resistance genes from these bacteria that are co-extracted during antibiotic preparations, allowing widespread environmental exposure to genetic elements capable of imparting antibiotic resistance [15]. Although this theory may lack rigorous scientific support, antibiotics clearly exert a selective pressure on bacteria, quickening evolution and allowing concurrent adjustments in genetics and metabolism to produce multidrug resistant phenotypes [16,17].

#### 2.1.2. Acquired Resistance

The bacteria‘s ability to not only survive in the presence of antibiotics but also to acquire resistance under antibiotic selective pressures assumes that a threshold concentration of antibiotics is necessary to both induce and sustain resistance genotypes [18,19].

### 2.2. Mechanisms

Regardless of if it is innate or acquired, several different mechanisms of resistance exist.

#### 2.2.1. Horizontal Gene Transfer (HGT)

**Figure 2:**
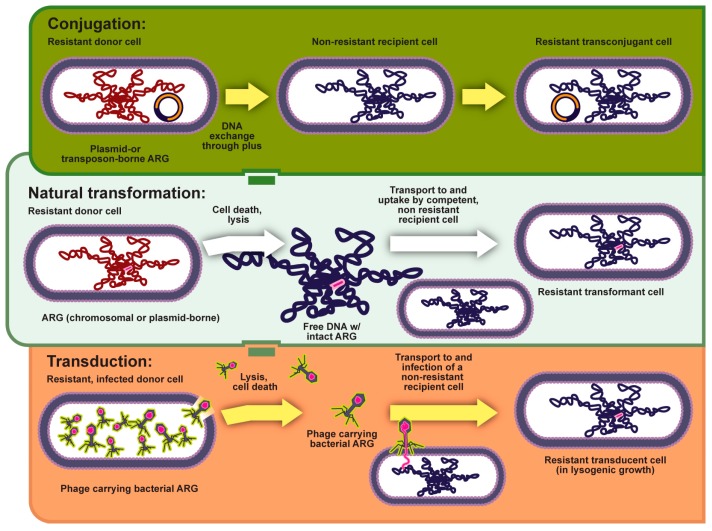
Horizontal gene transfer can commonly occur through conjugation and natural transformation. Additionally, it may occur through transduction, where resistance is transmitted via bacteriophage.

The majority of acquired antibiotic resistance is propagated through horizontal or lateral gene transfer between bacteria often due to the polymicrobial nature of infections and proximity of pathogens [20,21,22]. Lateral gene transfer can occur in one of three ways (1) uptake of environmental DNA [23]; (2) infection by bacteriophages [24]; or (3) recombination/exchange of plasmids (Figure 2) [25,26,27]. However, such a transfer of genetic material does not come without an evolutionary cost [26,28]. To minimize this evolutionary cost bacteria have developed several genetic strategies such as plasmids [29,30], transposons, gene clusters [30], and operons [6]. While all of these strategies represent complexity when treating antibiotic resistance, a combination of an operon or cluster surrounded by mobile genetic elements is perhaps the most ominous, allowing genetic elements that confer antibiotic resistance to move as an unaltered block [5,21,30]. The ability to shuffle genetic material between species may explain not only the transfer of antibiotic resistance but also the expansion of resistance beyond a single drug. High-level resistance often produces low-level resistance to an antibiotic in the same class as a byproduct [31].

#### 2.2.2. Elevated Mutation Rates

Despite the fitness costs, antibiotic pressure selects for bacteria that have a heightened mutation rate [11,21,32]. *Mycobacterium* spp. are capable of acquiring de novo resistance via mutation under inadequate treatment regimes [31,33,34,35,36] as are *Escherichia coli*/*Salmonella enterica* (fluoroquinones) [37,38,39] and *Pseudomonas aeruginosa* [31,40,41]. This increased mutation rate is typically conferred by alterations in the genes that constitute the mismatch repair (MMR) system (*mutS*, *mutL*, *mutH*, *mutT*, *mutY*, *mutM*, and *uvrD*) [42]. Mutations in the MMR system also increase the prevalence of genetic recombination, providing diversity to antibiotic resistance mechanisms [21]. Beyond heritable genetic changes to mismatch repair systems as well as mutations in gyrase and topoisomerase enzymes (quinolone resistance in *Samonella* [43]), antibiotics can also increase mutation rates via oxidative damage [44,45,46] and more broadly, the stress responses [44,47].

### 2.3. Adaptive Resistance

Acquired resistance often arises due to pressures from the surrounding microbiome; however, adaptive resistance is a reflection of the ecological niche of the microbe. It is increasingly evident that there are vast reservoirs of antibiotics in the environment capable of enriching antibiotic resistant pathogens [31,48,49]. Thus, adaptive resistance includes environmentally induced genetic changes such as biofilm and persister development, enzymatic driven antibiotic inactivation, changes in the antibiotic target, changes in cell permeability, and efflux pump regulation.

#### Drug Resistance in Biofilms

Amid the torrent of environmental stresses, it is thought that the majority of bacteria, particularly in the presence of a foreign body or under prolonged exposure to sub-inhibitory antibiotic concentrations, reside in surface adherent biofilms [50,51,52]. Biofilm formation occurs through a series of events coordinated through cell-cell communications (i.e., quorum sensing), as mediated by excreted autoinducers (i.e., small molecules in gram-negative bacteria [53,54] and peptides in gram-positive bacteria [55,56,57]). Signaling cascades initiate upon detection of a critical extra cellular concentration of autoinducer [4,21] and culminate in adhesion, metabolic changes, production of a protective glycocalyx, up-regulation of virulence, and decreased antibiotic susceptibility among other factors [21,58,59]. Interrupting bacterial adhesion prior to glycocalyx formation may prove an antibiotic strategy with efficacy analogous to those therapies designed to kill planktonic bacteria [60,61]. Importantly, once a surface adherent biofilm has been established therapies designed to kill planktonic bacteria are ineffective. The glycocalyx protects bacterial inhabitants from antibiotics, biocides, and other chemical or physical obstacles [62,63] and is recognized a key element in the persistence of infections [64]. The secretion of an extracellular glycoprotein matrix provides a protected ecological niche for the proliferation and the propagation of antibiotic resistance through the exchange of genetic material [65,66], allowing the accumulation of mutations and genetic elements that confer resistance over time [15,29,67]. These events have been thoroughly reviewed and are beyond the scope of this review [53,68]. Evidence holds that the development of biofilm is a major pathway to development of resistance.

Resistance conferred by a biofilm is likely not just a consequence of an encompassing glycocalyx but may also result from the underlying heterogeneous bacterial subpopulations. These sub-populations vary not only in their degree of antibiotic susceptibility but also the mechanism by which they achieve their states of antibiotic resistance [19,63]. Certain sub-populations of biofilm bacteria may produce enzymes that degrade antibiotics while other sub-populations have up-regulated efflux pumps [53]. The overall ecology of the biofilm community also imposes endogenous oxidative stress on its members, which consequently drives biofilm bacteria to a hyper-mutability state [47,69,70]. In addition to being capable of quickly acquiring a multidrug resistant phenotype, this combination of resistance mechanisms has lead scientists to describe biofilms as having enhanced resistance for a vast array of antibiotic classes [53,66]. Sub-populations can also be metabolically distinct, a fact that has critical implications for clinical treatment and the de novo development of resistance.

Bacterial persisters are a metabolically senescent sub-population within a biofilm. Although several good reviews describe aspects of persister cells in biofilms [19], briefly exploring the impact of metabolic senescence is useful to frame a discussion of therapeutic development to circumvent this resistance [71,72]. In general, the efficacy of most antibiotics is based on the metabolic activity of the bacteria (e.g., β-lactams that target cell wall synthesis in actively dividing *P. aeruginosa* [19,53,73] and quinolones that typically introduce nicks in actively replicating DNA [74]); therefore, non-growing or slow-growing cells are less susceptible to antibiotic effects, a fact that has been repeatedly demonstrated in vitro using bulk cultures forced into a prolonged stationary phase [75]. When cells are challenged with antibiotics during this stationary phase of slowed growth, they display a persistence phenotype, having increased survival when compared to cells in a logarithmic phase of growth under the same antibiotic pressure [19,73,76], giving rise to a property called—drug indifference [77].

### 2.4. Antibiotic Inactivation

A common mechanism of antibiotic resistance involves the enzymatic modification or destruction of the antibiotic [78,79,80]. Generally, four classes of antibiotic-modifying enzymes are known: hydrolases, group transferases, redox enzymes, and lysases [78]. Many of these are considered exotoxins and increase virulence while concurrently providing antibiotic resistance. Hydrolases, such as the host of amidases that cleave the β-lactam ring, cleave hydrolytically susceptible chemical bonds such as esters and amides, which are often essential for antibiotic biological activity. This is a highly popular strategy to engender antibiotic resistance and increased virulence as these water-soluble enzymes can be easily excreted from bacteria as a prophylactic counter measure, intercepting antibiotic molecules prior to contact with the bacteria [78,81]. The group transferases (e.g., acyltransferases [82], phosphotransferases [79], thioltransferases [83], nucleotidetransferases [84], ADP-ribosyltransferases [85], glycosyltransferases [86]) represent a broad and well-characterized class of bacterial enzymes that impose covalent modifications on antibiotics to inhibit antibiotic target binding [87]. Aminoglycoside resistance is often conferred on the basis of transferase (acetyltransferases, nucleotidyltransferases (adenylyltransferases), and phosphotransferases) prompted antibiotic inactivation [88,89].

Redox enzymes do not represent a widely recognized strategy for antibiotic modifications; however, there are some noted exceptions, the most extensively studied being tetracycline resistance endowed by the TetX enzyme [90]. Finally, lysases cleave carbon-carbon, carbon-oxygen, carbon-nitrogen, and carbon-sulfur bonds by a mechanism independent of either hydrolytic or oxidative cleavage. An example is Vgb, responsible for streptogramin resistance [91,92].

#### 2.4.1. Alteration of the Antibiotic Target

Antibiotic targets can be reprogrammed or camouflaged such as by factor-assisted protection of the drug target [80]. One such example is that of penicillin binding protein 2 (PBP2), which based on the horizontal gene transfer and acquisition of *mecA*, confers a low affinity binding of many β-lactam antibiotics such that the bacteria are able to continue cell wall biosynthesis unimpeded [93]. Modification of the antibiotics target site (e.g., methylation of rRNA) can also serve to reduce affinity with limited costs to fitness [80]. Additional molecular mechanisms of resistance were reviewed by Walsh in 2000, Blair in 2015, and Munita in 2016 [6,94,95].

#### 2.4.2. Changes in Cell Permeability and Efflux

Bacterial efflux pumps have evolved as a protective mechanism in both gram-positive and, particularly, gram-negative [30,80] bacteria to maintain cell homeostasis and communication by actively pumping out solutes, metabolites, quorum sensing molecules, and toxins, specifically antimicrobial compounds [30,53,93,96,97,98]. There are five families of bacterial drug efflux pumps: the ATP-binding cassette (ABC) superfamily [99], the major facilitator superfamily (MFS) [100], the multidrug and toxic compound extrusion (MATE) family [101], the small multidrug resistance (SMR) family (a subgroup of the drug/metabolite transporter superfamily [102]), and the resistance-nodulation-division (RND) superfamily [103,104]. However, multiple drug resistance can be acquired via up regulation in a single efflux pump, making this a highly effective system [30,105,106,107]. Li et al. evaluated a library of antimicrobial compounds, finding that all but 2 of the 33 compounds tested were susceptible to efflux by the endogenous multidrug efflux system in *E. coli* [108]. This lack of drug specificity facilitates the synergistic coupling of efflux up regulation with other resistance mechanisms, particularly changes in outer membrane permeability and will likely negatively impact drugs in development.

## 3. Virulence Mechanisms

The transmission of antibiotic resistance and virulence has many parallel mechanisms as outlined below.

### 3.1. Virulence Factors

Virulence encompasses not only the ability of bacteria to cause disease in the host (i.e., degree of pathogenicity) but also the ability of bacteria to infiltrate and colonize a host. In the late 1980s and early 1990s, virulence was thought of as a pathogen centered attribute and the concept of pathogenicity islands of genetic virulence factors was introduced in the late 1990s [109,110]. Genetic virulence factors can regulate physical attributes of the bacteria such as flagella, curli, fimbriae, adhesions, biofilm, [111,112,113,114] or biochemical factors, including host cell surface modifying enzymes, toxins, and antibiotics to provide a competitive advantage [115,116,117]. Genetically encoded antibiotic resistance in many aspects can be considered a subtype of virulence factors as they promote host pathogenesis, allowing persistent or chronic diseases [118]. Identifying pathogenic islands of virulence and antibiotic resistance genes along with their associated transcriptional and translational regulators is critical to recognize and assess druggable targets in the search for new tools in the antibacterial arsenal. Equally important in antibiotic drug discovery efforts is a consideration of virulence transmission, and the potential combinatorial approach with anti-virulence therapeutics.

#### 3.1.1. Adhesion Molecules

**Figure 3:**
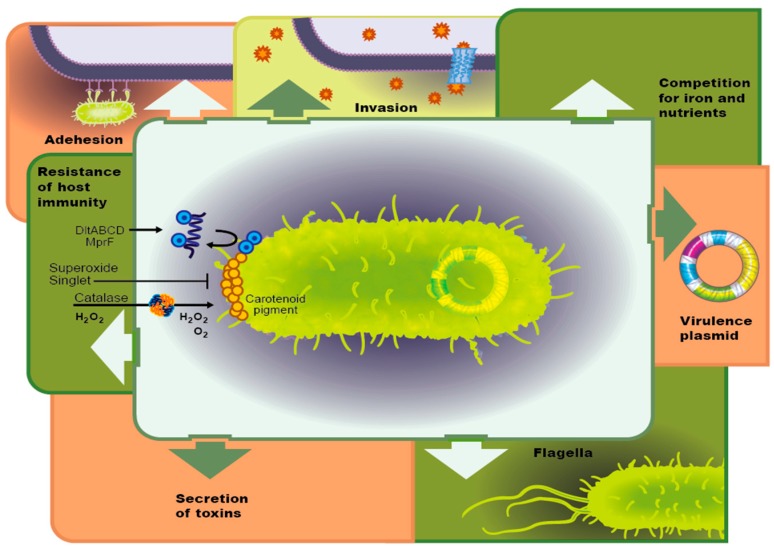
Summary of virulence factors commonly recognized.

The bacterial ability to adhere and form biofilms on biotic and abiotic surfaces causes an increase in virulence, as well as bacterial pathogenicity. Bacterial attachment is facilitated through cell surface organelles, such as flagella, pili, fimbriae, and curli (Figure 3) [119]. The assembly and regulation of flagella is genetically controlled through an operon (e.g., the FlhD/FlhC complex [120,121], which can be regulated by phosphorylation of OmpR and RcsB [122] or a two-component system [123,124]. Regardless of the mechanism, the upregulation of genetically controlled adhesion molecules also regulates biofilm development. Adhesion, quorum sensing and biofilm development have evolved as a complex mechanism by which bacteria can respond selectively to antibiotic pressure [122].

#### 3.1.2. Host Tissue Invasion

Invasion of bacteria into host cells is facilitated by upregulation in enzymes such as collagenase, hyaluronidase, and lecithinase that break down cell membranes. Alternatively, certain bacteria such as *Staphylococcus aureus* are able to infiltrate osteoblasts via a fibronectin bridge or collagen binding protein (Cna). Finally, bacteria can trigger a cell signal cascade through either a zipper or trigger mechanism by reorganizing the actin cytoskeleton of non-professional phagocytic cells [125].

#### 3.1.3. Competition for Resources and Iron

Iron acquisition has been related to virulence in a variety of bacterial pathogens [126]. In *Riemerella anatipestifer*, an infection that affects poultry, siderophore-interacting protein (SIP) was identified as necessary not only for iron acquisition but also for optimal virulence. A deletion of the *sip* gene resulted in decreased biofilm formation and a reduction in adherence to and invasion of Vero cells [127].

#### 3.1.4. Host Immune Evasion

The *Salmonella* plasmid virulence (*spv*) locus reduced the LD50 of *Salmonella enterica serovar typhimurium* in zebrafish larvae and inhibited neutrophils and macrophages, allowing bacterial replication to accelerate. Additionally, the *spv* locus was found to prevent autophagy of *S. typhimurium* by inhibiting the formation of the autophagosomes, leading to the supposition that the *spv* locus is involved in suppressing the innate immune response [128]. Alternatively, diversity in the glycosylated structure of siderophores has not only produced functional redundancy and specificity concurrently but has also served to avoid host defenses, such is the case with Lipocalin 2, which can sequester the siderophore, Enterobactin but not is glycosylated derivative Salmochelin [126]. Group B *Streptococcus* produces a phosphodiesterase that limits type I interferon to promote virulence [129,130]. Finally, a variety of bacterial pathogens have evolved molecular mimicry to hijack and manipulate the host’s inhibitory immune signaling as well as the ability to directly interact with host cytokines and other factors [131,132,133]. These represent just a small subset of bacterial strategies for immune evasion; bacteria have evolved a plethora of virulence factors that contribute to host immune evasion [134].

#### 3.1.5. Bacterial Toxin Secretion

Many bacteria secrete toxins based on upregulation of virulence genes. These can be classified as either endotoxins, which are the lipid portion of lipopolysaccharide that is released upon cell death, or exotoxin, which are secreted into the extracellular space. Recently, a study of *P. aeruginosa* isolated from children with cystic fibrosis had increased expression of the virulence genes *toxA*, *lasB* and *exoS*. These genes are involved in both chronic and acute forms of the disease and are involved in tissue damage and establishment of the biofilm [112]. *S. aureus* is also known for its ability to secrete a host of toxins to aid in host tissue infiltration and acquire nutrients, as discussed above. Additionally, these pervasive pathogens also produce cytolytic toxins among a host of others, including hemolysin, leukotoxin, exfoliative toxin and enterotoxin that aid in bacterial growth [135]. This common mechanism of bacterial virulence should be investigated as a source of new druggable targets.

#### 3.1.6. Bacterial Motility

Bacterial pathogens use a variety of different motility modes, including swimming, twitching, and swarming. Increased and variable motility cannot only allow a pathogen to change physical location but is also associated with over expression of a large number of virulence genes. *P. aeruginosa* swarming was associated with increased expression of virulence genes, such as type III secretion and iron transport as well as an increase in antibiotic resistance to polymyxin B, gentamicin, and ciprofloxacin [136].

### 3.2. Virulence Plasmid

Plasmids are an adaption bacteria utilize to transfer genetic material between bacteria of the same species, as well as between different bacterial species. Many plasmids in pathogenic bacteria only encode virulence genes, antibiotic resistance genes, or genes required to activate virulence genes on the chromosome. Shiga-toxin producing *E. coli* (STEC) are associated with food borne illness. One of the most important serotypes, O157:H7, possesses a large virulence plasmid—pO157. This highly studied plasmid is believed to encode the virulence genes *ehxA* (enterohemolysin) [137], *etpC* to *etpO* (Type II Secretion System) [138], *espP* (serine protease) [139], *katP* (catalase-peroxidase) [139], *toxB* (a potential adhesion) [140], *stcE* (zinc metalloprotease) [141], and *ecf* (*eae* conserved fragment) [142] (For a review, see [143]). One study investigated 22 STEC and 4 non STEC strains, isolated from humans, pigs, and a variety of other environmental samples, comparing whole genome sequences to identify virulence and novel antibiotic resistance plasmids. A total of 39 novel plasmids were identified, many of them contained virulence genes from pathogenic *E. coli* groups, including STEC. In addition, 8 of these plasmids contained 29 antibiotic resistance genes encoding resistance to these major antibiotic groups: aminoglycosides, carbapenems, penicillins, cephalosporins, chloramphenicol, dihydrofolate reductase inhibitors, sulfonamides, tetracyclines and resistance to heavy metals. Broad-spectrum antibiotic resistance could be conferred because many of the plasmids contained more than one antibiotic resistance gene. This study showed that virulence plasmids can be found in many *E. coli* strains or serotypes, which were isolated from diverse locations, demonstrating that plasmids with multiple virulence and antibiotic resistance genes can be easily spread amongst the *E. coli* species [144].

## 4. Regulation of Virulence and Antibiotic Resistance

### 4.1. Types of Regulation

**Figure 4:**
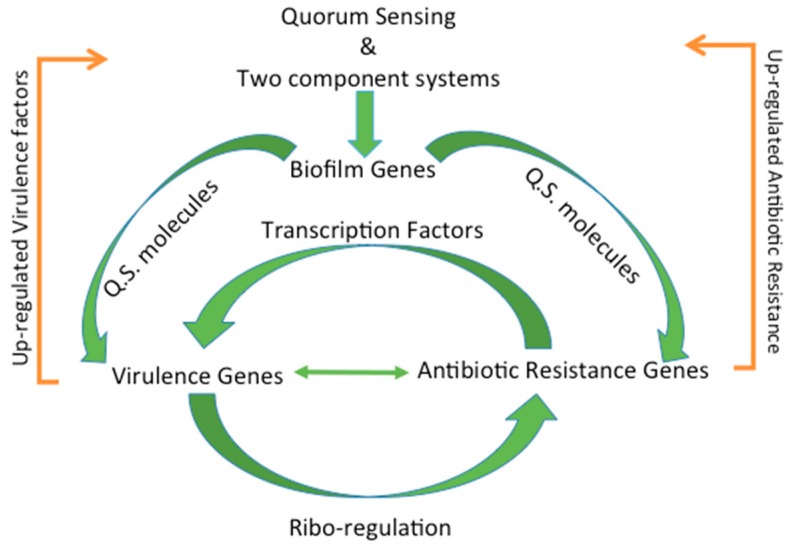
Common regulatory mechanisms act as a connection between virulence and antibiotic resistance. Quorum sensing and two component systems upregulate biofilm genes, which in turn upregulate quorum sensing molecules that in turn influence virulence and antibiotic resistance genes. Up-regulated virulence and antibiotic resistance genes serve to upregulate quorum sensing and two component systems and complete the cycle.

The regulation of virulence and antibiotic resistance genes is very complex. Traditionally this regulation is thought of as separate events; however, the genetic regulation is intertwined and connected (Figure 4). Often times the regulation of virulence genes can influence the expression of antibiotic resistance genes and vice versa. Unfortunately, regulation of these genes is impacted by a host of environmental factors that can directly and indirectly affect gene expression. Below, the most common forms of gene regulation, including post-transcriptional modifications (e.g., methylation, etc.), ribo-regulation, an environmental sensing, stress response (e.g., (p)ppGpp), multi-networks of regulation, and biofilm formation through quorum sensing are reviewed.

### 4.2. Transcription Factors

Transcription factors can regulate gene expression directly or indirectly through global gene regulators in both virulence and antibiotic resistance genes. Regulation of virulence genes where the transcription factors bind directly to the promoter region has been seen in Enterohemorrhagic *Escherichia coli* (EHEC) where the transcription factors Cra, KdpE, and FusR bind directly to the *ler* region on the locus of enterocyte effacement (LEE) pathogenicity island in order to control the gene expression [145]. The global transcriptional regulator H-NS, has been shown that EHEC *hns* deletion mutants showed lower survival rates and a higher bacterial burden in the gut of infected BALB/c mice. In comparison the wild type EHEC strains were more virulent and had a higher ability to colonize the gut of the infected BALB/c mice, this was found to be especially true when antibiotics were not administered, leading to the conclusion that H-NS interacts with the gut microbiota of the mice in order to cause virulence [146]. Alternatively, PrfA, a member of the cyclic AMP transcriptional regulators that appears to be a master virulence regulator in *Listeria monocytogenes* as it binds DNA as dimers in gene promoters to activate transcription [147].

Regulation of antibiotic resistance genes through transcription factors have been observed in methicillin resistant *S. aureus* (MRSA), particularly strains with intermediate level resistance to vancomycin (VISA). VISA is facilitated by the transcription factors *yycH* and *yycI*, which was shown to down-regulate the two-component regulator WalRK when the two genes were knocked out. Additionally, down-regulation of the cell wall hydrolase genes *atlA* and *sle1* was seen to cause impaired autolytic function of the deletion mutants when compared to the wildtype. This reduction in WalRK expression leads to decreased ability for cell wall turnover, which ultimately leads to a decreased effect of vancomycin [148]. Another example is the identification of Ms4022 a novel TetR transcriptional regulator in *Mycobacterium smegmatis* has been shown to activate the expression of several transport drugs that have affects the strains resistance to drugs. When Ms4022 was overexpressed in the wildtype strain of *M. smegmatis,* its growth was inhibited, but the strain’s resistance to the anti-tuberculosis drug rifampicin was enhanced. Alternatively, deletion of Ms4022 showed an increased susceptibility to the drug rifampicin. Ms4022 was found to directly regulate seven transport related genes that are thought to result in the resistance this strain has for rifampicin [149]. Several other genetic regulators—*relA*, *spot*, *brlR*, and *crc* (catabolite repression regulator) have been investigated to increase antibiotic tolerance in *P. aeruginosa* in response to either ciprofloxacin or tobramycin [150,151]. Other regulatory genes fall in the oxidative stress, hypoxia (i.e., Anr regulon), osmotic stress, growth arrest (RpoS), and quorum sensing pathways, which have been shown to play a role in antibiotic sensitivity [150]. The precise transcriptional regulation of antibiotic resistance is also dependent on the type of antibiotic.

### 4.3. Post-Transcriptional Modifications

Post-translational modifications are a well recognize mode of regulation; however, regulation of virulence and antibiotic resistance are also tied to post-transcriptional regulations. Post-transcriptional modifications, which act as a quality control mechanism to produce stable RNA transcripts, occur much more commonly in prokaryotes than is reported [152]. In *Salmonella enterica* the translation of the virulence associated effector protein AvrA was completely abolished, while transcription was unaffected when the genes CsrA/CsrB were deleted. The expression of *avrA* is regulated by a post transcriptional modification induced by the effective concentration of CsrA, which is achieved by sequestering of CsrB [153]. The post-transcriptional regulator Crc has been shown in recent research to modulate *P. aeruginosa* antibiotic susceptibility and virulence. One study showed *crc* deficient mutants of *P. aeruginosa* were found to secrete lower levels of virulence factors, including toxins and proteins necessary for cytolysis; which is hypothesized to be the reason the mutant was also had reduced cytotoxicity in comparison to the wildtype strain. Lowered amounts of ToxA, virulence determinant exotoxin A, were also found in the vesicle-free secretome of *P. aeruginosa crc* deficient mutant when compared to the wildtype [154]. *Mycobacterium tuberculosis* is a significant health threat and the leading cause of death related to antimicrobial resistance. Recently, resistance to a potent pyrido-benzimidazole antimicrobial, 14, was investigated revealing that each mutation that conferred resistance fell in the putative DNA-binding and dimerization region of *rv2887*, a transcriptional repressor of the multiple antibiotic resistance regulator (MarR) family. The mutations in *rv2887*, led to increased expression of *rv0560c*, a methyltransferase that N-methylates 14 to abolish its activity [155]. Alternatively, since the production and use of bacterial virulence factors in *Yersinia* spp. Is energetically costly, their expression is tightly regulated [156]. These post transcriptional methods involve binding of mRNA with a variety of molecules, including regulatory RNA-binding proteins such as YopD and the low calcium response secretion chaperone, small regulatory and non-coding RNAs such as Ysr35, thermosensors such as *yscW-IcF* intergenic region, and RNases [156].

While the presence of post-translational modifications has been shown to increase virulence factors, the lack of post-transcriptional modifications to rRNA has been shown to cause an increase in antibiotic resistance to some antibiotics. For example, the *tylA* encodes a methyltransferase that methylates the 16S and 23S ribosomal RNA, however loss of methylation ability results in increased resistance to capreomycin and viomycin. On the other hand, post-transcriptional modifications have also been shown to increase susceptibility of a strain. Many post-transcriptional modifications are focused on the peptidyl transferase, which is the mode of action of many antibiotics. One study found that 8 genes in *E. coli* involved in rRNA modification at the peptidyl transferase, when knocked out had increased the susceptibility of *E. coli* to peptidyl transferase inhibiting antibiotics [157]. 

### 4.4. Ribo-Regulation

Clearly, RNA binding elements are responsible for post-transcriptional modifications, but RNA species are also responsible for ribo-regulation of virulence and antibiotic resistance genes. In termination-based ribo-regulators, the presence of antibiotic allows either read through or premature termination, ultimately, providing for production of antibiotic resistance [158]. Such mechanisms have been identified in *Bacillus subtilis* (*vmlR* and *bmrB*), *L. monocytogenes* (*rli53* and *rli59* which control expression of *lmo 0919* and *lmo1652*). Early termination by the riboswitch occurs in the absence of the antibiotic, but in the presence of lincomycin causes a conformational change in the transcriptional terminator to induce the full expression of the antibiotic resistance gene [158]. Certain termination-based regulators act in *cis* with the promoter. Cis-regulation of RNA elements are known as riboswitches or attenuators, which are often present in the gene’s 5′ UTR, can cause early termination of transcription by direct ligand binding in order to control bacterial gene expression. In the presence of different metabolite concentrations, the riboswitch structure is altered upon ligand binding, which allows the full expression of the gene due to destabilization of the terminator. Theophylline-sensitive synthetic riboswitches were used to induce gene expression of -galactosidase reporter in the intracellular pathogen *Francisella.* Riboswitching has also been shown to control the expression of FTN_0818 under nutrient limited environments and is required for replication in macrophages. FTN_0818 mutants growth was shown could be rescued in minimal media by riboswitch mediated control of FTN_0818 [159]. In another study, it was found that termination riboswitches regulated numerous antibiotic resistance genes in pathogenic bacteria, but when the antibiotic was present in the cell it allowed for read through or complete expression of the gene. *L. monocytogenes* has 55 recognized riboswitches that respond to 13 distinct ligands to control virulence and antibiotic resistance [147]. A complete description of these riboswitches is beyond the scope of this review; however, the reader is directed to a 2015 review by Oliva et al on riboswitches that impact virulence and metabolism [160]. Suffice it to say that riboswitches are essential to gene regulation involved in bacterial physiology and virulence, and may provide potential targets for new drug therapies.

### 4.5. Environmental Flux Sensing

A central principle in effective regulation of both antibiotic resistance and virulence is the ability to sense and respond to the bacteria’s external environment, specifically, the presence and concentration of ambient antibiotic. The vancomycin-responsive, histidine kinase in *Streptomyces coelicolor* or the LiaRS cell envelope damage sensing system of *B. subtilis* exemplifies the importance of this ability [161]. The system in *B. subtilis* is regulated by the two component system BceRS and its associated ABC-transporter BceAB [161]. Over 200 Bce-like systems have been identified [162]. Transcriptome profiling, which predicts expression patterns of pathogens in the presence of antimicrobial agents, recently identified virulence and resistance mechanisms of *S. aureus* in the presence of increasing concentrations of ramoplanin. Ramoplanin resistant strains of *S. aureus* acquired resistance to multiple antibiotics and with corresponding increased resistance regulation of biotin synthesis, the citric acid cycle, riboflavin biosynthesis, peptidoglycan biosynthesis, folic acid biosynthesis, heme biosynthesis and amino acid metabolism [163]. The disruption of metabolic processes then indirectly affects virulence factors such as biofilm formation. Alternatively, the presence of antibiotics can alter regulation of pneumococcal bacteriocins production [164].

### 4.6. Stress Response

Multiple stress response pathways have been identified as key players in the regulation of antibiotic resistance and virulence. Environmental factors, such as oxygen concentration, can impact the effect transcription factors on virulence and antibiotic resistance genes. It was shown that Cra strongly activates expression of the LEE pathogenicity island under aerobic conditions, while under these same conditions FusR represses expression [165]. In *S. aureus* the two-component system SrrAB is required for anaerobic respiration. SrrAB has been shown to down-regulate the regulatory RNA *agr*-RNAIII, which aids in the excretion of the virulence factors: serine protease and α-hemolysin [166]. It also has been shown to increase expression of the *ica* operon, which increases extracellular polysaccharide [163].

Alternatively, the stress response has been shown to play a key role in beta-lactam resistance in all tested strains of MRSA [167]. Guanosine pentaphosphate and guanosine tetraphosphate have also been shown critical for the bacterial stress response [168]. In another study, the effect of low and high external calcium concentrations on virulence factors on *Pseudomonas aeruginosa* PAO1 revealed mutations in the gene *carP*, which is part of the CarSR two-component system, that resulted in reduced motility, pyocyanin production, and tobramycin sensitivity [166]. Additionally, the carbon storage regulatory system (Csr) has been found to be related to regulation of virulence in response to stress [169] while the stress-responsive, alternative sigma factor, σ^B^, activates a variety of virulence genes in the intestine in *L. monocytogenes* [147].

### 4.7. Complex Multi-Regulation Networks

Regulation via genetic transcriptional factors represents a complex link between virulence and antibiotic resistance. A mutation in the *typA* gene in *P. aeruginosa* lowered virulence, thereby evading phagocytosis, increasing attachment, and enhancing biofilm formation. Additionally, this mutation, which initially may be viewed as a fitness cost led to increased antibiotic resistance, and down regulation of the type III secretion system [145]. Conversely, Bartoli et al reported that *Pseudomonas viridiflava*, a phytopathogenic bacterium, has an inverse correlation between antibiotic resistance and pathogenicity, indicating that fitness may be species specific [170]. *TypA* has also been shown to play a major role in regulation of virulence factors in enteropathogenic *E. coli*, including flagella, type III secretion system, LEE pathogenicity island, and *espC* pathogenicity island [165]. Recently, a transposon based genetic screen performed in *Acinetobacter baumannii* identified a subset of 30 transcription factors that were not only necessary for proliferation in the host but also were present in conditions of antibiotic resistance and environmental stress, highlighting the existence of a link between virulence and antibiotic resistance [171]. *A. baumannii* is not the only bacteria to exhibit such a connection, Skurnik et al reported that mutation in *P. aeruginosa*’s OprD entry channel, which provides resistance to carbapenem antibiotics, also led to a dramatic increase in the ability of *P. aeruginosa* to colonize the mucosa in a mouse model, disseminate to the spleen, and prove cytotoxic to murine macrophages [172].

### 4.8. Biofilm Formation through Quorum Sensing

This link between virulence and antibiotic resistance often resides within the formation of a biofilm community structure. Biofilm formation is an essential part of pathogenicity (or a combination of virulence and antibiotic resistance); thus, the formation is highly regulated by a complex network of transcriptional regulation. Formation of biofilms occur in several steps with each step typically associated with a specific cell surface organelle [146,173]. Reversible attachment, where the bacterium may loosely attach to a surface and detach again is aided by flagella, fimbriae, and pili. Once the cells begin to produce adhesions and some extracellular polymer matrix the cells are considered irreversibly attached to the surface. During the maturation phase, the attached cells begin to proliferate and produce an extracellular polysaccharide matrix, which helps define the three-dimensional structure of the biofilm [146,149]. The initiation of biofilm formation not only begins with quorum sensing, which may be considered a virulence regulator, but the resistance of the biofilm structure itself may also be considered a regulator of both virulence and antibiotic resistance. Once a biofilm is established, it can activate additional virulence genes within the biofilm again through quorum sensing or two-component systems. In *L. monocytogenes*, the VirR/S controls expression of 17 genes, including resistance to a variety of antibiotics and food preservatives [147]. Eliciting a response that upregulates genes involved in competence, conjugation, motility, sporulation, biofilm formation, and many other virulence factors [159]. Furthermore, in addition to biofilms being inherently more resistant to antibiotics, they also facilitate the transfer and spread of antibiotic resistance and virulence due to the close proximity of the cells. This process is accelerated when put under environmental stress, such as in the presence of an antimicrobial. Finally, the conversion of certain cells to persisters and regulation of virulence is associated with a significant enrichment of genes regulated by MyfR [174].

## 5. Biofilms Enhance the Transmission of Virulence and Antibiotic Resistance

### 5.1. Biofilm Formation

The Centers for Disease Control (CDC) has revealed that the majority of bacteria occur in a biofilm, or a community of bacteria protected by a secreted exopolysaccharide, slimy matrix. Interestingly, the apparent form of bacterial altruism, leads to community behavior, including synchronized regulation of virulence genes. Biofilms are already up to 1000 times more resistant to antibiotics when compared to their free-floating, planktonic counterparts. Furthermore, this coordinated community, protected by a slimy matrix called the glycocalyx also facilitates the spread of antibiotic resistance [62,64,175,176]. Antibiotic resistant strains of *P. aeruginosa* were shown to have enhance biofilm production and a coordinating regulatory protein to switch on and off antibiotic resistance in *P. aeruginosa* [177].

Although single species biofilms have proven deadly, as evidenced by extensive study of *P. aeruginosa* single species biofilms in patients, particularly children, with cystic fibrosis [178], it is now widely believed that biofilms usually are made up of multiple species of bacteria, which have (1) proven more difficult to treat and (2) caused more persistent infections than mixed species of planktonic bacteria (e.g., *P. aeruginosa*, *Pseudomonas protegens*, *and Klebsiella pneumonia*). These multispecies bacterial communities also had slower development trends and distinct structures in comparison to the single species biofilms [179]. These in vitro observations have been born out in vivo. In an in vivo study, a mixed species biofilm of *P. aeruginosa* and *S. aureus* caused longer wound healing times and increased host immune response in a rabbit model [180,181]. Similar results were not obtained from single species infecting pathogens. Regardless of the homogeneity in biofilm composition, it is clear that the establishment of a biofilm enhances virulence.

### 5.2. Bacterial Communication

Communication amongst bacteria is essential for many cellular functions, such as biofilm formation, sporulation, competence, antibiotic resistance, and regulation of a variety of other virulence factors [182,183,184]. Bacteria communicate either through (1) quorum sensing and (2) two component systems, which are directed and deliberate forms of communication of bacteria. 

Quorum sensing cell-to-cell communication coordinates cellular actions based on bacterial density. In quorum sensing mechanisms, individual bacteria secrete chemical signal molecules, autoinducers (AI). As bacterial density increases, the concentration of AI in the immediate environment also increases and interacts with cell signal receptors on surrounding bacteria [57,185,186]. Acylated homoserine lactone autoinducers mediate communication between gram-negative bacteria. Alternatively, two additional autoinducers; autoinducer 1 (AI-1) and autoinducer 2 (AI-2), mediate species-specific communication molecules and interspecies communication molecules, respectively [187,188,189,190]. Other secreted peptides, also known as autoinducing peptides, facilitate gram-positive bacteria quorum sensing [191]. In addition to using quorum sensing as a means to communicate for biofilm formation, bacteria can also use quorum sensing to increase virulence. In *Acidovorax citrulli*, quorum sensing molecules enhanced virulence of the strain by influencing genes that control cellular attachment and biofilm formation [192]. Enhancing virulence genes that increase biofilm formation can facilitate the exchange of antibiotic resistance genes.

Virulence can also be enhanced via two-component bacterial communication systems [193]. Two-component systems work by binding an external signaling molecule to a histidine kinase receptor protein that causes a phosphorylation cascade event [194,195]. WalKR, a two-component system in *S. aureus* that controls multiple cellular functions, is essential to virulence and is being investigated as a potential target to combat staph infections [196]. Regardless of the specific bacterial communication mechanism, it is clear that communication enhances virulence and may provide new druggable targets with little chance for bacterial resistance to arise due to (1) lack of selective life or death pressure and (2) few fitness advantages.

### 5.3. Horizontal Transfer of Virulence Genes

Similar to the transfer of antibiotic resistance genes, virulence genes can be acquired via a variety of mechanisms, including spontaneous mutations and deliberate exchange of genes through horizontal gene transfer. Horizontal gene transfer allows the transmission of genes both between individuals of a single species and between multiple species. Of course, such a transfer is facilitated by proximity; thus, when bacterium convert from planktonic, free floating individuals, to a sessile collective, transfer via plasmids is hastened (Figure 2 and Figure 3) [62,197,198]. In a study of the role of plasmid-mediated horizontal gene transfer of antibiotic resistance, samples of the gut microbiome from hospitalized patients and healthy volunteers, 46 genes were identified. Multiple commonalities were identified among the individual patients. Surprisingly, one antibiotic resistance gene was also identified in a single healthy volunteer. Unfortunately, the study was unable to identify the source of resistance (i.e., nosocomial or normal flora) [199]. In addition to horizontal gene transfer due to proximity, other environmental factors (e.g., acidic or alkaline environments) also play a role in the transfer of genes, as evidenced by the increase in tetracycline resistance genes in wastewater sludge when grown under acidic environmental conditions, revealing an increased potential for a horizontal gene transfer of tetracycline resistance genes located on a plasmid [200].

## 6. Conclusions

Combating increasing antibiotic resistance is one of the most important problems that plagues our society today. Unfortunately, approaching this grand challenge as a single etiology limits the solutions. A distinctly new approach considering the interdependence of both the regulation and transmission of antibiotic resistance and virulence must be taken to make significant progress in this global problem. The connection between the underlying genetic elements that confer antibiotic resistance and increased virulence is intimately tied to the ability of bacteria to communicate through quorum sensing and two component systems both directly and indirectly. The exchange, transfer, expression, and regulation of these genes are further facilitated not only in cases where both genetic elements are present on the same plasmid, but also when bacteria occur in a biofilm state. Increased virulence may naturally evolve in response to or concurrently with increased antibiotic resistance; thus, in controlling the spread of antibiotic resistance, we must also control the spread of virulence. This may be accomplished via new combination anti-virulence strategies that do not impose life or death selective pressures [201]. By understanding regulation of virulence and antibiotic resistance, particularly how their regulation can influence each other; this will allow us to provide more specific and directed drug treatments.
